# Supplementary material for: Can the triglyceride-glucose index predict the risk of stroke? A meta-analysis of high-quality studies with 12.8 million participants
Source: PeerJ. 2025 Sep 9;13:e19994. doi: 10.7717/peerj.19994 (PMC12428551; doi:10.7717/peerj.19994)
Supplement: Supplemental Information 2 [file peerj-13-19994-s002.docx]

Supplementary Table 1**:** Detailed description of the search strategy

| **PubMed** | |
| --- | --- |
| #1 | ‘triglyceride-glucose index’[MeSH Terms] |
| #2 | ‘triglyceride and glucose index’ OR ‘TyG index’ OR ‘triglyceride glucose index’ OR ‘triacylglycerol glucose index’ |
| #3 | ‘Stroke’[MeSH Terms] |
| #4 | ‘stroke’ OR ‘Cerebrovascular Accident’ OR ‘CVA’ OR ‘Brain Vascular Accident’ OR ‘Cerebrovascular Disease’ |
| #5 | #1 OR #2 |
| #6 | #3 OR #4 |
| #7 | #5 AND #6 |
| **Embase** | |
| #1 | ‘triglyceride-glucose index’: ab,ti |
| #2 | ‘triglyceride and glucose index’ OR ‘TyG index’ OR ‘triglyceride glucose index’ OR ‘triacylglycerol glucose index’ |
| #3 | ‘Stroke’/exp OR 'stroke' |
| #4 | ‘stroke’ OR ‘Cerebrovascular Accident’ OR ‘CVA’ OR ‘Brain Vascular Accident’ OR ‘Brain Vascular Accidents’ OR ‘Cerebrovascular Disease’ |
| #5 | #1 OR #2 |
| #6 | #3 OR #4 |
| #7 | #5 AND #6 |
| **Scopus** | |
| #1 | ‘triglyceride-glucose index’ TITLE-ABS-KEY-AUTH |
| #2 | ‘triglyceride and glucose index’ OR ‘TyG index’ OR ‘triglyceride glucose index’ OR ‘triacylglycerol glucose index’ |
| #3 | ‘Stroke’ TITLE-ABS-KEY-AUTH |
| #4 | ‘stroke’ OR ‘Cerebrovascular Accident’ OR ‘CVA’ OR ‘Brain Vascular Accident’ OR ‘Brain Vascular Accidents’ OR ‘Cerebrovascular Disease’ |
| #5 | #1 OR #2 |
| #6 | #3 OR #4 |
| #7 | #5 AND #6 |
| **Web of Science** | |

(((((triglyceride-glucose index) OR (triglyceride and glucose index)) OR (TyG index)) OR (triglyceride glucose index)) OR (triacylglycerol glucose index)) AND (((((Stroke) OR (Cerebrovascular Accident)) OR (CVA)) OR (Brain Vascular Accident)) OR (Cerebrovascular Disease))

| **CENTRAL** |
| --- |

(((((triglyceride-glucose index) OR (triglyceride and glucose index)) OR (TyG index)) OR (triglyceride glucose index)) OR (triacylglycerol glucose index)) AND (((((Stroke) OR (Cerebrovascular Accident)) OR (CVA)) OR (Brain Vascular Accident)) OR (Cerebrovascular Disease))
