# Supplementary material for: Can the triglyceride-glucose index predict the risk of stroke? A meta-analysis of high-quality studies with 12.8 million participants
Source: PeerJ. 2025 Sep 9;13:e19994. doi: 10.7717/peerj.19994 (PMC12428551; doi:10.7717/peerj.19994)
Supplement: Supplemental Information 3 [file peerj-13-19994-s003.docx]

Supplementary Table 2: List of excluded studies with reasons and references

| Excluded study | Reason |
| --- | --- |
| Lopez-Jaramillo et al[1] | Including prior stroke |
| Huang et al[2] | Including prior stroke |
| Wu et al[3] | Cross-sectional study |
| Jiang et al[4] | Cross-sectional study |
| Wang et al[5] | No stroke outcomes |
| Chen et al[6] | No stroke outcomes |
| Mirashafiei et al[7] | No stroke outcomes |
| Zhang et al[8] | On hypertensive population |
| Hu et al[9] | On hypertensive population |
| Esin et al[10] | On patients with patent foramen ovale |
| Ayca et al[11] | On patients with patent foramen ovale |
| Tian et al[12] | Overlapping data |
| Wang et al[13] | Overlapping data |
| Cui et al[14] | Overlapping data |
| Huang et al[15] | Overlapping data |
| Jung et al[16] | Overlapping data |
| Wang et al[17] | Overlapping data |
| Wang et al[18] | Overlapping data |
| Xu et al[19] | Overlapping data |
| Zhao et al[20] | Overlapping data |
| Cui et al[21] | Overlapping data |
| Xia et al[22] | Overlapping data |
| Xu et al[23] | Overlapping data |
| Liang et al[24] | Overlapping data |
| Yu et al[25] | Overlapping data |
| Wu et al[26] | Overlapping data |
| Feng et al[27] | Overlapping data |

References

1 Lopez-Jaramillo P, Gomez-Arbelaez D, Martinez-Bello D, Abat MEM, Alhabib KF, Avezum Á, et al. Association of the triglyceride glucose index as a measure of insulin resistance with mortality and cardiovascular disease in populations from five continents (PURE study): a prospective cohort study. Lancet Heal Longev. 2023;4(1):e23–33.

2 Huang Q, Yin L, Liu Z, Wei M, Feng J, Huang Q, et al. Association of novel lipid indicators with the risk of stroke among participants in Central China: a population-based prospective study. Front Endocrinol (Lausanne). 2023;14(October):1–12.

3 Wu M, Li C, Yu Y, Zeng L, Qiu Y, Liu J, et al. Association between the triglyceride-glucose (TyG) index and stroke risk in Chinese normal-weight adults: a population-based study. Diabetol Metab Syndr. 2024;16(1):1–11.

4 Jiang M, Wu H, Zhang H, Su F, Cao L, Ren X, et al. Association between the Triglyceride-Glucose Index and the Risk of Large Artery Atherosclerotic Stroke. Int J Clin Pract. 2022;2022. DOI: 10.1155/2022/5191581

5 Wang S, Zhang X, Keerman M, Guo H, He J, Maimaitijiang R, et al. Impact of the baseline insulin resistance surrogates and their longitudinal trajectories on cardiovascular disease (coronary heart disease and stroke): a prospective cohort study in rural China. Front Endocrinol (Lausanne). 2023;14(December):1–12.

6 Chen F, Pan Y, Liu Z, Huang R, Wang J, Shao J, et al. Impact of Visit-to-Visit Triglyceride-Glucose Index Variability on the Risk of Cardiovascular Disease in the Elderly. Int J Endocrinol. 2022;2022. DOI: 10.1155/2022/5125884

7 Mirshafiei H, Darroudi S, Ghayour-Mobarhan M, Esmaeili H, AkbariRad M, Mouhebati M, et al. Altered triglyceride glucose index and fasted serum triglyceride high-density lipoprotein cholesterol ratio predict incidence of cardiovascular disease in the Mashhad cohort study. BioFactors. 2022;48(3):643–50.

8 Zhang N, Chi X, Zhou Z, Song Y, Li S, Xu J, et al. Triglyceride-glucose index is associated with a higher risk of stroke in a hypertensive population. Cardiovasc Diabetol. 2023;22(1):1–8.

9 Hu L, Bao H, Huang X, Li M, Cheng X, Zhou W, et al. Relationship Between the Triglyceride Glucose Index and the Risk of First Stroke in Elderly Hypertensive Patients. Int J Gen Med. 2022;15(December 2021):1271–9.

10 Esin F, Ince HS, Aktürk S, Citekci FT, Celik A, Kocabas U, et al. The Relationship Between the Triglyceride-Glucose Index and Cryptogenic Stroke in Patients with Patent Foramen Ovale. Clin Appl Thromb. 2024;30. DOI: 10.1177/10760296241301411

11 Ayça B, Yıldız C, Yüksel Y, Katkat F, Arpaç A, Çağlar FNT, et al. Evaluation of Triglyceride Glucose Index in Patients with Patent Foramen Ovale Who Experienced Cryptogenic Stroke. J Clin Med. 2024;13(23):1–11.

12 Tian X, Zuo Y, Chen S, Meng X, Chen P, Wang Y, et al. Distinct triglyceride-glucose trajectories are associated with different risks of incident cardiovascular disease in normal-weight adults. Am Heart J. 2022;248:63–71.

13 Wang A, Tian X, Zuo Y, Chen S, Meng X, Wu S, et al. Change in triglyceride-glucose index predicts the risk of cardiovascular disease in the general population: a prospective cohort study. Cardiovasc Diabetol. 2021;20(1):1–9.

14 Cui H, Liu Q, Wu Y, Cao L. Cumulative triglyceride-glucose index is a risk for CVD: a prospective cohort study. Cardiovasc Diabetol. 2022;21(1):1–9.

15 Huang Z, Ding X, Yue Q, Wang X, Chen Z, Cai Z, et al. Triglyceride-glucose index trajectory and stroke incidence in patients with hypertension: a prospective cohort study. Cardiovasc Diabetol. 2022;21(1):1–10.

16 Jung MH, Yi SW, An SJ, Yi JJ, Ihm SH, Han S, et al. Associations between the triglyceride-glucose index and cardiovascular disease in over 150,000 cancer survivors: a population-based cohort study. Cardiovasc Diabetol. 2022;21(1):1–10.

17 Wang X, Feng B, Huang Z, Cai Z, Yu X, Chen Z, et al. Relationship of cumulative exposure to the triglyceride-glucose index with ischemic stroke: a 9-year prospective study in the Kailuan cohort. Cardiovasc Diabetol. 2022;21(1):1–9.

18 Wang A, Wang G, Liu Q, Zuo Y, Chen S, Tao B, et al. Triglyceride-glucose index and the risk of stroke and its subtypes in the general population: an 11-year follow-up. Cardiovasc Diabetol. 2021;20(1):1–9.

19 Xu W, Zhao H, Han X, Liu J, Li H, Sun J, et al. Relationship between early-onset stroke and triglyceride-glucose index among young Chinese adults. Lipids Health Dis. 2023;22(1):1–11.

20 Zhou H, Ding X, Lan Y, Chen S, Wu S, Wu D. Multi-trajectories of triglyceride-glucose index and lifestyle with Cardiovascular Disease: a cohort study. Cardiovasc Diabetol. 2023;22(1):1–11.

21 Cui H, Liu Q, Zhao Z, Ma X. Interacting and joint effects of triglyceride-glucose index and blood pressure on cardiovascular diseases risk: a prospective cohort study. Diabetol Metab Syndr. 2024;16(1):1–9.

22 Xia X, Chen S, Tian X, Xu Q, Zhang Y, Zhang X, et al. Association of triglyceride-glucose index and its related parameters with atherosclerotic cardiovascular disease: evidence from a 15-year follow-up of Kailuan cohort. Cardiovasc Diabetol. 2024;23(1):1–11.

23 Xu F, Feng Y, Zhong X. Higher triglyceride‑glucose index is associated with increased risk of stroke among middle-aged and elderly Chinese: a national longitudinal study. Sci Rep. 2024;14(1):1–11.

24 Liang W, Ouyang H. The association between triglyceride-glucose index combined with obesity indicators and stroke risk: A longitudinal study based on CHARLS data. BMC Endocr Disord. 2024;24(1):234.

25 Yu Y, Meng Y, Liu J. Association between the triglyceride-glucose index and stroke in middle-aged and older non-diabetic population: A prospective cohort study. Nutr Metab Cardiovasc Dis. 2023;33(9):1684–92.

26 Wu Y, Yang Y, Zhang J, Liu S, Zhuang W. The change of triglyceride-glucose index may predict incidence of stroke in the general population over 45 years old. Cardiovasc Diabetol. 2023;22(1):1–9.

27 Feng G, Yang M, Xu L, Liu Y, Yu J, Zang Y, et al. Combined effects of high sensitivity C-reactive protein and triglyceride–glucose index on risk of cardiovascular disease among middle-aged and older Chinese: Evidence from the China Health and Retirement Longitudinal Study. Nutr Metab Cardiovasc Dis. 2023;33(6):1245–53.
