# Supplementary material for: Can the triglyceride-glucose index predict the risk of stroke? A meta-analysis of high-quality studies with 12.8 million participants
Source: PeerJ. 2025 Sep 9;13:e19994. doi: 10.7717/peerj.19994 (PMC12428551; doi:10.7717/peerj.19994)
Supplement: Supplemental Information 4 [file peerj-13-19994-s004.docx]

Raw data

| Study | Categorical | Continuous |
| --- | --- | --- |
| Hong 2020 | 1.260 (1.234, 1.287) |  |
| Zhao 2021 | 1.95 (1.34–2.82) |  |
| Cho 2022 | 1.253 (1.167, 1.346) |  |
| Liu Q 2022 | 1.22 (1.11–1.33) | 1.12 (1.08–1.17) |
| Liu L 2022 | 1.402 (0.853–2.305)) | 1.196 (0.917–1.561) |
| Che 2023 | 0.99 (0.90–1.10) | 0.98 (0.95–1.02) |
| Muha-mmad 2023 | 1.30 (1.18, 1.44) |  |
| Wan 2023 | 1.44 (1.10–1.88) | 1.39 (1.19–1.61) |
| Wang 2023 | 1.254 (1.014–1.552) | 1.321 (1.161–1.504 |
| Li-1 2024 | 1.49 (1.11, 1.99) | 1.32 (1.08, 1.61) |
| Li 2024 | 1.46 (1.16–1.85) | 1.14 (1.06–1.23) |
| Rafiee 2024 | 1.45 (0.96–2.19) |  |
| Yao 2024 | 1.43 (0.94) | 1.08 (0.93) |

Meta-regression moderators

| Study | Sample size | Age (y) | Male (%) | DM (%) | HT (%) | DL (%) | TyG cut-off | Stroke incidence (%) | F/U (y) | NOS score |
| --- | --- | --- | --- | --- | --- | --- | --- | --- | --- | --- |
| Hong 2020 | 5593134 | 52 | 50.5 | 3.7 | 26.9 | 11.2 |  | 1.59 | 8.2 | 9 |
| Zhao 2021 | 11777 | 53 | 40.9 |  |  |  | 9.14 | 5.74 | 6 | 9 |
| Cho 2022 | 6675424 | 31 | 59.6 | 0 | 6.9 |  | 8.34 | 0.13 | 7.4 | 9 |
| Liu L 2022 | 6095 | 48.7 | 49.1 | 0 | 46.5 |  | 8.76 | 2.5 | 10.6 | 8 |
| Liu Q 2022 | 96541 | 51 | 79.6 | 9 | 43.3 | 0.75 | 9.05 | 5.3 | 10.3 | 9 |
| Che 2023 | 403335 | 56.2 | 44.8 | 3.8 | 13.9 | 6.7 | 9.07 | 1 | 8.1 | 9 |
| Muha-mmad 2023 | 32920 | 45 | 67.5 | 2.5 | 5.5 |  | 4.74 | 13.3 | 16.9 | 9 |
| Wan 2023 | 42651 | 55.7 | 40.3 | 10.2 | 50 | 34.6 | 9.02 | 1.6 | 4.7 | 9 |
| Wang 2023 | 10132 | 54.1 | 46 | 9 | 33 |  |  | 9 | 26.6 | 9 |
| Li-1 2024 | 3534 | 59 | 40.2 | 19 | 69.1 |  | 9.04 | 9 | 10 | 9 |
| Li 2024 | 10569 | 59 | 47.1 | 6.1 | 39.7 | 9.8 | 9.07 | 7.1 | 7 | 9 |
| Rafiee 2024 | 5432 | 50.7 | 48.8 | 8.4 | 27.8 | 87.2 |  | 3.16 | 11.2 | 9 |
| Yao 2024 | 6890 | 57 | 38.6 | 5.7 | 53.8 |  | 9.06 | 3.89 | 4.3 | 9 |
